# Unlocking Alzheimer’s Disease: The Role of BDNF Signaling in Neuropathology and Treatment

**DOI:** 10.1007/s12017-025-08857-x

**Published:** 2025-05-17

**Authors:** Saad Misfer Alqahtani, Hayder M. Al-kuraishy, Ali I. Al Gareeb, Ali K. Albuhadily, Athanasios Alexiou, Marios Papadakis, Loah R. Hemeda, Safaa A. Faheem, Gaber El-Saber Batiha

**Affiliations:** 1 Department of Pathology, College of Medicine, Najran University, Najran, Saudi Arabia; 2 Department of Clinical Pharmacology and Medicine, College of Medicine, Mustansiriyah University, Baghdad, Iraq; 3 Department of Clinical Pharmacology and Medicine, Jabir Ibn Hayyan Medical University, Al-Ameer Qu./Najaf - Iraq Po. Box (13), Kufa, Iraq; 4 University Centre for Research & Development, Chandigarh University, Chandigarh-Ludhiana Highway, Mohali, Punjab India; 5 Department of Research & Development, Funogen, 11741 Athens, Greece; 6 University Hospital Witten-Herdecke, University of Witten-Herdecke, Heusnerstrasse 40, 42283 Wuppertal, Germany; 7 Department of Medicinal Chemistry, Faculty of Pharmacy, Beni-Suef University, Beni-Suef, 62514 Egypt; 8 Department of Pharmacology and Toxicology, Faculty of Pharmacy, Egyptian Russian University, Cairo-Suez Road, Badr City, 11829 Cairo Egypt; 9 Department of Pharmacology and Therapeutics, Faculty of Veterinary Medicine, Damanhour University, Damanhour, 22511 AlBeheira Egypt

**Keywords:** Brain-derived neurotrophic factor, Cholinergic neurons, Neurodegenerative diseases, Alzheimer’s disease, Statins

## Abstract

Alzheimer’s disease (AD) remains one of the most debilitating neurodegenerative disorders, with its pathological hallmark being progressive cognitive decline and memory loss. Recent research has illuminated the crucial role of the brain-derived neurotrophic factor (BDNF) in the central nervous system (CNS), highlighting its impact on neurogenesis, synaptic plasticity, and neuronal survival. Dysregulation of the BDNF signaling axis, particularly the imbalance between its precursor form and mature BDNF, is strongly implicated in the pathophysiology of AD. This review explores the molecular mechanisms through which BDNF modulates AD neuropathology and presents novel therapeutic strategies to activate BDNF signaling. We focus on the potential of BDNF activators, such as TrkB agonists and mimetic molecules, to restore synaptic function and ameliorate cognitive deficits in AD. Furthermore, we examine the challenges in translating these findings into clinical practice, including issues with blood–brain barrier penetration and the need for precise receptor targeting. The review emphasizes the therapeutic potential of repurposed drugs, including statins and metformin, in enhancing BDNF signaling and offers new insights into the future of AD treatment. Ultimately, this work provides a compelling argument for BDNF-based therapies as a promising avenue for mitigating the cognitive decline associated with Alzheimer’s disease, signaling a hopeful direction for future research and clinical trials.

## Introduction

Brain-derived neurotrophic factor (BDNF) is a particular protein that plays several roles in the central nervous system (CNS), including neurogenesis, synaptic plasticity, neuronal development and differentiation, and synaptogenesis (Ali et al., 2024a, 2024b, 2024c; Okezie et al., 2022). BDNF is synthesized chiefly in CNS areas such as the thalamus, hippocampus, and limbic system (Azman & Zakaria). In addition, BDNF is also synthesized peripherally by the immune cells, skeletal muscles, vascular endothelium, and platelets (Murawska-Ciałowicz et al., 2021). Peripheral BDNF, however, cannot pass through the blood–brain barrier (BBB) (Diniz et al., 2020). The frontal cortex, hippocampus, striatum, hypothalamus, and midbrain are the primary locations of central BDNF expression (Dou et al., 2022). In the endoplasmic reticulum (ER), BDNF synthesis begins as pre-proBDNF, which is subsequently transferred to the Golgi apparatus to produce proBDNF. Plasmin and furin convert proBDNF to mature BDNF in the trans-Golgi system (Banerjee & Shenoy, 2023). Moreover, proBDNF is also catalyzed by plasmin and metalloproteinases to form truncated BDNF, whose physiological function is unknown (Keifer, 2021). Most proBDNF (85%) is converted to mature BDNF, though this pathway is distributed in AD leading to the reduction in the formation of mature BDNF. It has been confirmed that mature BDNF was reduced by 23% compared to 30% of proBDNF (Peng et al., 2005).

It has been shown that proBDNF concentration is higher during childhood due to brain development, though BDNF concentration is higher in adulthood to preserve cognitive function (Cui et al., 2024). Of note, different stages in the life of a dendritic spine, including formation, maturation, and plasticity, are strictly regulated by proBDNF and BDNF. Long-term synaptic plasticity, including long-term potentiation (LTP) and long-term depression (LTD), represents the cellular mechanism underlying learning and memory processes (Menshanov et al., 2015; Sun et al., 2021). Both LTP and LTD are associated with structural changes in dendritic spines. BDNF signaling modulates dendritic spine morphology and is required for the induction and maintenance of LTP and learning and memory processes. The BDNF/TrkB signaling impairments are highly correlated with cognitive impairment and dendritic spine changes during aging. While the p75 neurotrophin receptor (p75^NTR^) also binds the mature BDNF, albeit at a lower affinity, it preferentially mediates the action of proBDNF. However, proBDNF/p75^NTR^ opposes the BDNF/TrkB ligand–receptor interaction that induces LTD (Cumming, 2022). In the developing brain, mature BDNF and its precursor proBDNF exhibit prosurvival and proapoptotic functions, respectively. However, it is still unknown whether mature BDNF or proBDNF is a major form of neurotrophin expressed in the immature brain and during normal brain development. Findings from pre-clinical study found that both proBDNF and mature BDNF were expressed abundantly in the rat brainstem, hippocampus, and cerebellum between embryonic day 20 and postnatal day 8. The levels of mature BDNF to proBDNF ratios negatively correlated with the expression of active caspase-3 across brain regions. The immature cortex was the only structure, in which proBDNF was the major product of *BDNF* gene, especially in the cortical layers 2–3. And only in the cortex, the expression of BDNF precursor positively correlated with the levels of active caspase-3 (Menshanov et al., 2015). These findings suggest that proBDNF alone may play an important role in the regulation of naturally occurring cell death during cortical development. It has been shown that postnatal proBDNF played an essential role in synaptic and cognitive functions. Furthermore, the expression of proBDNF compared to mature BDNF during brain development (Sun et al., 2021).

**Fig. 1:**
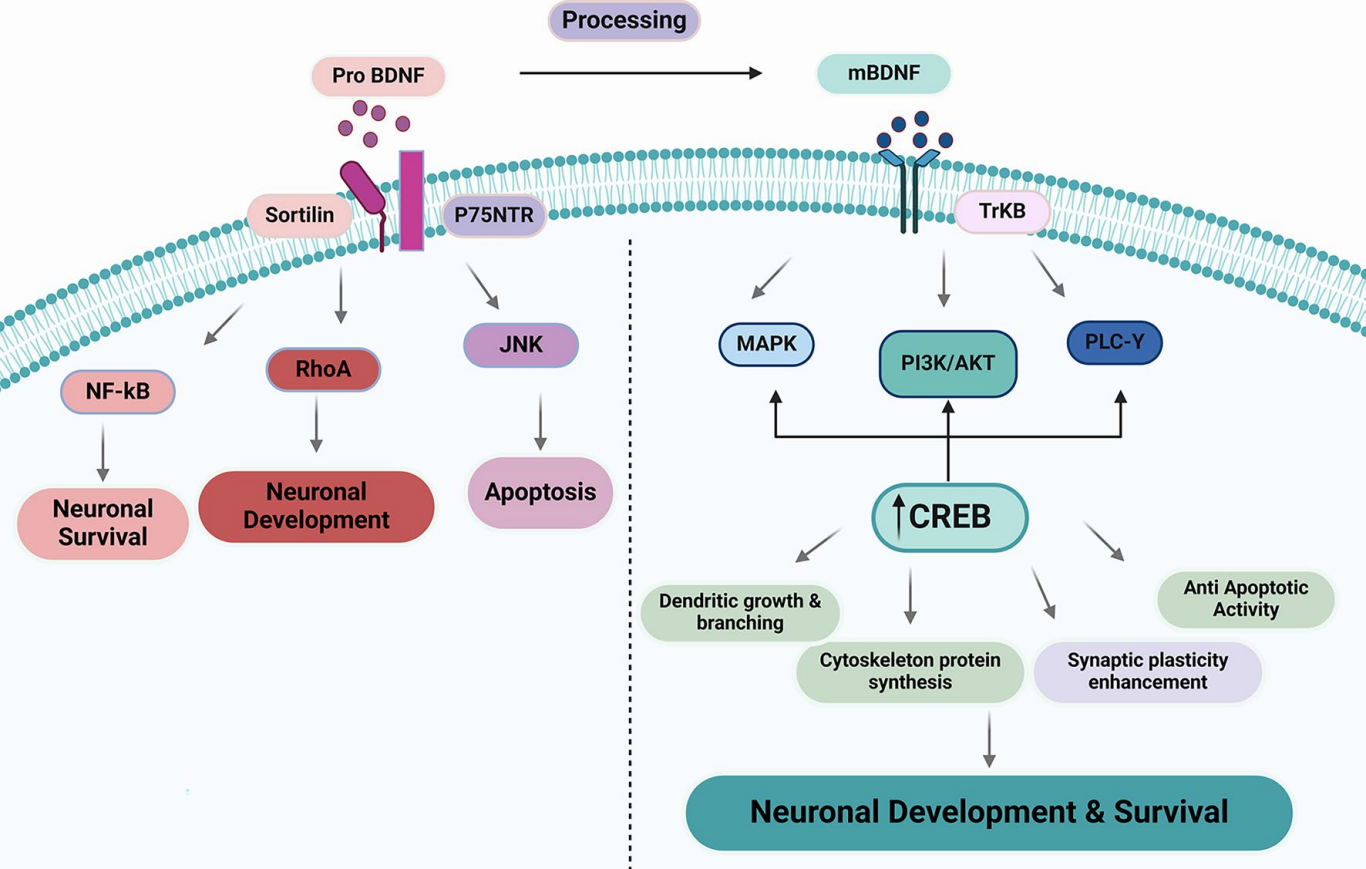
proBDNF/BDNF signaling: proBDNF is converted to the BDNF, proBDNF activates p75^NTR^ with sortilin receptor complex, which causes the expression of c-Jun N-terminal kinase (JNK), Ras homolog family member A (RhoA), and nuclear factor kappa B (NF-κB), leading to neuronal survival, neuronal development, and induction of apoptosis. By activating the TrkB receptor, BDNF stimulates the activation of protein kinase B (Akt), phospholipase C gamma (PLC-γ), mitogen-activated protein kinase (MAPK), phosphoinositol triphosphate kinase (PI3K), and protein kinase B (MAPK). Additionally, BDNF enhances the expression of cAMP-response element-binding protein (CREB), which promotes synaptic plasticity, dendritic growth, and apoptosis inhibition

While proBDNF activates p75^NTR^, BDNF carries out its biological action via activating the tropomyosin receptor kinase type B (TrkB) (Yang et al., 2022; Zagrebelsky & Korte, 2024). proBDNF/BDNF axis exerts different physiological effects, for example, proBDNF/p75^NTR^ with sortilin receptor form a proBDNF/p75^NTR^ sortilin complex, resulting in various signaling pathways being activated, which causes neuronal apoptosis and regulation of neuronal survival and differentiation (Al-Yozbaki et al., 2020). However, BDNF/TrkB, via activation of many signaling pathways, promotes synaptic plasticity, dendritic growth, and other neuroprotective effects (Numakawa & Odaka, 2021). The exact role of BDNF regarding its intracellular localization in neurons is controlled by numerous mechanisms, including the use of distinct promoters, mRNA and protein transport, and regulated cleavage of proBDNF to mature BDNF. Sortilin is an intracellular chaperone that binds to the prodomain of BDNF to traffic it to the regulated secretory pathway. However, sortilin binds to numerous ligands and plays a major role in mannose 6-phosphate receptor-independent transport of lysosomal hydrolases utilizing motifs in the intracellular domain that mediate trafficking from the Golgi and late endosomes. Sortilin is modified by ectodomain shedding, although the biological implications of this are not known (Evans et al., 2011). It has been shown that ADAM10 is the preferred protease to cleave sortilin in the extracellular stalk region, to release the ligand binding sortilin ectodomain from the transmembrane and cytoplasmic domains. Both sortilin and BDNF are trafficked to and degraded by the lysosome in neurons, and this is dependent upon the sortilin cytoplasmic tail. Indeed, expression of the sortilin ectodomain, which corresponds to the domain released after shedding, impairs lysosomal targeting and degradation of BDNF (Evans et al., 2011). These verdicts characterize the regulation of sortilin shedding and identify a novel mechanism by which sortilin ectodomain shedding acts as a regulatory switch for delivery of BDNF to the secretory pathway or to the lysosome, thus modulating the bioavailability of endogenous BDNF (Fig. 1).

It has been shown that dysregulation of the proBDNF/BDNF axis is intricate in the emergence of several neuropsychiatric conditions, including depression (Pettorruso et al., 2024). In addition, dysregulation of the proBDNF/BDNF axis is connected to the emergence of certain neurodegenerative diseases. Therefore, in various stages of neurodegenerative disorders involving Parkinson’s disease (PD) and AD, dysregulation of BDNF signaling is delayed until the development of advanced neurodegeneration (Faria et al., 2014a, 2014b). It was suggested that the increased BDNF levels were due to a compensatory mechanism to fight early neurodegeneration or to the activation of immune cells. As the disease advances, these compensatory processes may fail, resulting in lower BDNF levels in the peripheral blood and brain (Faria et al., 2014a, 2014b). BDNF prevents neuronal death by activating inositol triphosphate three kinase (IP3K) and Aκt (Pettorruso et al., 2024). BDNF and related molecular signaling decrease in neurodegenerative diseases and aging (Zarneshan et al., 2022). For instance, research on AD and PD in both humans and animals has shown a decline in the serum levels of BDNF (Huang et al., 2021; Yi et al., 2021). Reduction of BDNF expression increases the buildup of α-Syn in the dopamine neurons of the substantia nigra pars compacta (SNpc), aggravating the neuropathology of PD (Cao et al., 2022). Notably, tyrosine hydroxylase in the striatum is activated by BDNF, which maintains the dopaminergic neurons in the SNpc (Cao et al., 2022; Mohamed et al., 2023).

Also, local silencing of the *BDNF* gene triggers the gradual degeneration of the SNpc’s dopaminergic neurons in mice (Meka et al., 2015). Interestingly, BDNF signaling through inhibition of oxidative stress prevents glutamate-induced neurotoxicity in PD and the final phase of amyotrophic lateral sclerosis (ALS) (Jin, 2020; Pradhan et al., 2019). Furthermore, in AD patients, dysregulation of BDNF signaling is associated with cognitive decline (Yan et al., 2021). Currently, there is no effective treatment in the management of AD. Thus, searching for novel therapeutic strategies for managing AD is recommended. Therefore, BDNF signaling activators may ameliorate AD neuropathology and cognitive impairment (Gerenu et al., 2017). Consequently, this review aims to discuss the possible role of BDNF in AD neuropathology and how BDNF activators control AD neuropathology.

## The Putative Role of BDNF in AD

Considerable evidence indicates that brain BDNF signaling through the TrkB receptor deteriorates in the AD brain at an early stage of the disease (Lin et al., 2022). Human genetic and experimental animal studies suggest that declined BDNF levels are associated with synaptic and neuronal loss and cognitive impairment with aging and AD. Still, there is little evidence that BDNF signaling would play a significant role in the disease-specific amyloid or tau pathology (Schueller et al., 2020; Yasutake et al., 2006). Finding an effective treatment for chronic neurodegenerative disorders still represents an unmet goal. There is substantial evidence that such disorders represent a combination of genetic determinants and failure of neuroprotective mechanisms, sparking a wider degree of interest in shedding light on the cellular changes responsible for these devastating disorders. Because of their role in the survival or differentiation of developing neurons and the recent discovery of their importance in regulating synaptic plasticity during adulthood, neurotrophic factors have been suggested as essential contributors to the etiology of neurodegenerative disorders (Schueller et al., 2020; Yasutake et al., 2006). AD is a complex, chronic, devastating disease that affects the expression of BDNF, a molecule that plays a pivotal role in synaptic plasticity and cognition (Nasrolahi et al., 2022). From these findings, it appears clear that BDNF is implicated in the mechanism of action of drugs that improve cognitive deficits in animal models of AD and AD patients.

### AD Neuropathology

**Fig. 2:**
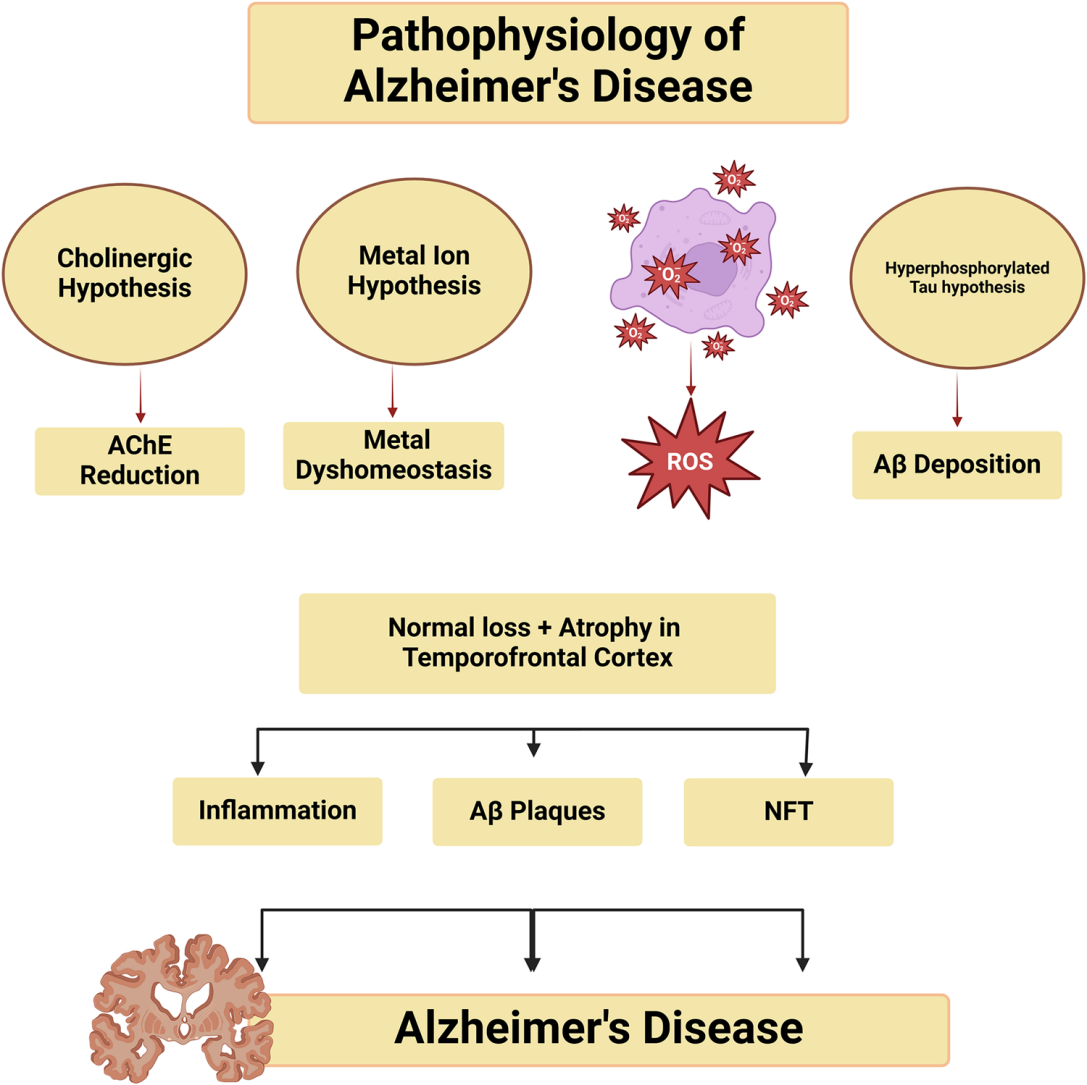
Pathophysiology of AD: cholinergic dysfunction, metal ion dyshomeostasis, amyloid beta (Aβ), tau protein hyperphosphorylation, and associated oxidative stress contribute in neuronal loss mainly in temporofrontal cortex. These neuropathological changes contribute in the accumulation of Aβ and hyperphosphorylated tau protein to form amyloid plaques and neurofibrillary tangles (NFTs), and the development of AD

AD is an aging-related chronic illness that causes progressive neuronal degeneration, mainly in the hippocampus and cerebral cortex, leading to amnesia and impaired cognition. AD is the most common neurological disease worldwide, affecting about 55 million people. An increased risk of AD is connected with an aging populace (Al-Kuraishy et al., 2023c). After the age of 65, women are more likely than males to have AD (Al-Kuraishy et al., 2023). Progressive accumulation of amyloid beta (Aβ) extracellularly and accumulation of mutant hyperphosphorylated tau protein intracellularly, which results in neurofibrillary tangles (NFTs), are the causes of AD neuropathology. AD has multiple causes that are influenced by environmental and genetic factors (Al-Kuraishy et al., 2023b). Research indicates that 95% of AD cases are sporadic, resulting from various conditions (Wang et al., 2023). However, because hereditary variables play a complex role in AD neuropathology, less than 5% of AD cases are familial (Al-Kuraishy et al., 2023d). Early onset AD development in familial AD is frequently distinguished from delayed onset AD development in sporadic AD (Al-Kuraishy et al., 2023d). Numerous mutations affecting the amyloid precursor protein (APP) gene cause an excess of amyloid protein beta (Aβ) in familial AD (Al-Kuraishy et al.). Furthermore, the apolipoprotein E4 (ApoE4) gene mutation is complex in AD neuropathology due to the buildup of Aβ and limits its clearance (Alsubaie et al., 2022; Kazim & Iqbal, 2016; Liu et al., 2010). Despite these discoveries, the basic mechanisms underlying the neuropathology of AD remain largely conjectural (Al-Kuraishy & Al-Gareeb, 2019; Alkuraishy et al., 2018; Al-Kuraishy et al., 2021, 2022c, 2022a, 2022b, 2020c, 2020a, 2020b, 2022c, 2022a, 2022b; Alorabi et al., 2022; Alsubaie et al., 2022; Hasan Khudhair et al., 2022). In addition, Aβ-neuroinflammation, oxidative damage, and mitochondrial abnormality contribute to AD progression (Ali et al., 2024a, 2024b, 2024c; Al-Kuraishy et al., 2020a, 2020b, 2020c) (Fig. 2).

### Role of BDNF in AD

Research has demonstrated the critical role that BDNF plays in the continuity and functionality of cholinergic nerve cells in the basal forebrain bundle (Orciani et al., 2022). Notably, cholinergic neurons in the basal forebrain bundle are distorted in AD, leading to memory loss and impaired cognition (Lin et al., 2022). Various investigations clarified that AD’s pathophysiology is linked to BDNF signaling disruption (Schueller et al., 2020; Yasutake et al., 2006). BDNF, the most widely distributed neurotrophin in the CNS, plays several pivotal roles in synaptic plasticity and neuronal survival. BDNF has a neuroprotective role against the pathogenesis of AD by reducing the expression and the activity of β-secretase, thereby inhibiting the amyloidogenic pathway and reducing neurotoxic Aβ formation in transgenic mice (Evans et al., 2011). In addition, BDNF enhances the expression and the activity of α-secretase with subsequent production of the neuroprotective soluble APP alpha (sAPPα) in transgenic mice (Evans et al., 2011).

As a consequence, BDNF became a key target in the physiopathology of several neurological and psychiatric diseases. Altered levels of BDNF in the circulation and the CSF in AD patients predict future cognitive decline in healthy older subjects (Schueller et al., 2020; Yasutake et al., 2006). Compared to other neurotrophic factors, such as ciliary neurotrophic factor (CNTF), which improves synaptic plasticity and cognitive impairment in animals, it has severe adverse outcomes. BDNF is more effective than CNTF in ameliorating cognitive impairment (Nasrolahi et al., 2022).

In addition, plasma levels of BDNF are reduced in AD patients or patients with cognitive impairment (Gezen-Ak et al., 2013; Nasrolahi et al., 2022; Shimada et al., 2014; Yasutake et al., 2006). Conversely, other research revealed no connection between AD patients’ plasma BDNF levels (Kim et al., 2017; Ng et al., 2019; Qin et al., 2017), which might be due to methodological biases (Balietti et al., 2018). Although the reasons for such different findings are unclear, methodological issues will likely be involved. The heterogeneity of participant recruitment criteria and the lack of control of variables that influence circulating BDNF levels regardless of dementia, such as depressive symptoms, medications, lifestyle, lack of overlap between serum and plasma, and experimental aspects are likely to bias results and prevent study comparability (Balietti et al., 2018). Despite these results, numerous pre-clinical and clinical investigations found a link between BDNF levels and AD neuropathology. A recent cross-sectional study illustrated that BDNF is higher in late-onset AD and lower in early-onset AD than controls (Wang et al., 2023), suggesting that expression of BDNF in plasma is altered at the clinical stage of AD.

Interestingly, serum BDNF level is regarded as a potential biomarker of AD neuropathology. Serum BDNF levels were lower in patients with AD and moderate cognitive dysfunction than in standard controls, according to case–control research involving 23 patients with AD, 22 patients having moderate cognitive dysfunction, and 21 standard controls (森友紀子, 2021). In addition, the serum level of BDNF was associated with the level of CSF Aβ and medial temporal lobe atrophy in AD (森友紀子, 2021). Similarly, in patients with AD, a decrease in blood BDNF level is more closely linked to cognitive impairment compared to vascular dementia (Yasutake et al., 2006). Therefore, the reduction of blood BDNF level is thought to be an early AD diagnostic biomarker and can be used to differentiate it from vascular dementia. Conversely, Faria et al. (Mayara Chaves Faria et al., 2014a, 2014b) discovered that AD patients had higher serum BDNF levels than healthy controls as a compensatory mechanism to limit further neurodegeneration. However, this small sample size and the fact that most AD patients did not fit the full criteria may not give concert evidence regarding this claim. Moreover, apathy in AD patients may limit physical exercise, a potent activator for increasing peripheral BDNF (Ribeiro et al., 2021). In addition, peripheral BDNF is reduced in aging, which is commonly associated with AD risk (Molinari et al., 2020). Therefore, aging and muscle atrophy in AD may affect the circulating level of BDNF.

According to a meta-analysis and systematic review, AD patients who are in their later stages typically have significantly lower serum BDNF levels (Ng et al., 2019). However, Gezen-Ak et al. (Gezen-Ak et al., 2013) highlighted that the level of BDNF is dropped in AD individuals with both early and late onset due to a progressive accumulation of Aβ within brain neurons, and associated neurodegeneration could be the possible mechanism for the reduction of BDNF levels. Interestingly, Aβ suppresses cAMP-response element-binding protein (CREB) expression (Amidfar et al., 2020). Also, Aβ selectively increases mRNA levels for the truncated TrkB isoforms without affecting TrkB-FL mRNA levels and triggers a calpain-mediated cleavage on TrkB-FL receptors in postmortem human brain samples. Furthermore, Aβ impairs BDNF function in a calpain-dependent way, as assessed by the inability of BDNF to modulate neurotransmitter release from hippocampal nerve terminals and LTB in hippocampal slices (A. Jerónimo-Santos et al., 2015a, 2015b). These findings suggest that Aβ-induced calpain activation leads to TrkB cleavage and impairment of BDNF neuromodulatory actions. Interestingly, Aβ can be produced by oligodendrocytes, the myelinating glia of the CNS that may affect the expression of neuronal BDNF (Sasmita et al., 2024), signifying the neuroprotective effects of oligodendrocytes against the development and progression of AD.

Therefore, CSF BDNF level is expected to be reduced in patients with AD due to neurodegeneration. According to Laske et al. (Laske et al., 2007), AD patients have lower CSF levels of BDNF than healthy controls due to progressive brain neurodegeneration, which is also present in patients with normal-pressure hydrocephalus. However, CSF BDNF level was correlated with serum BDNF level (Laske et al., 2007). Furthermore, there was no difference in the CSF level of BDNF between AD patients and those with depressive disorders and frontotemporal dementia (Blasko et al., 2005). The CSF level of BDNF was shown to be lowered and linked with a decrease in the expression of BDNF in the neocortex and hippocampal regions, according to a comprehensive review and meta-analysis of postmortem investigations (Du et al., 2018).

Findings from clinical studies observed that ApoE4 carriers had progressive cognitive impairment and low BDNF expression in AD patients (Lim et al., 2015). ApoE4 augments histone deacetylase expression in human neurons, leading to the suppression of BDNF expression. Histone deacetylase six increases in AD brains, mainly in the hippocampus, leading to low BDNF-induced cognitive impairment (Sen et al., 2015). Thus, ApoE4-induced Aβ expression can inhibit neuronal BDNF signaling in AD. Moreover, proBDNF signaling is also dysregulated, leading to the proBDNF/BDNF axis deregulation in AD (Haydar M Al-Kuraishy et al., 2023). It has been observed that the proBDNF expression is reduced by 40% in the parietal cortex of patients with advanced AD compared to the controls (Al-Kuraishy et al., 2023b). ProBDNF processing and recycling are of interest mainly in the astrocytes (Al-Kuraishy et al., 2023d). It has been illustrated that Aβ disturbs the functional activity of astrocytes (Al-Kuraishy et al., 2023e; 2023f), which might cause impairment of proBDNF processing. Brain atrophy in AD reduces the neuronal capacity for proBDNF processing, increasing proBDNF, which induces more neuronal apoptosis and brain atrophy (Ferrero et al., 2018).

**Fig. 3:**
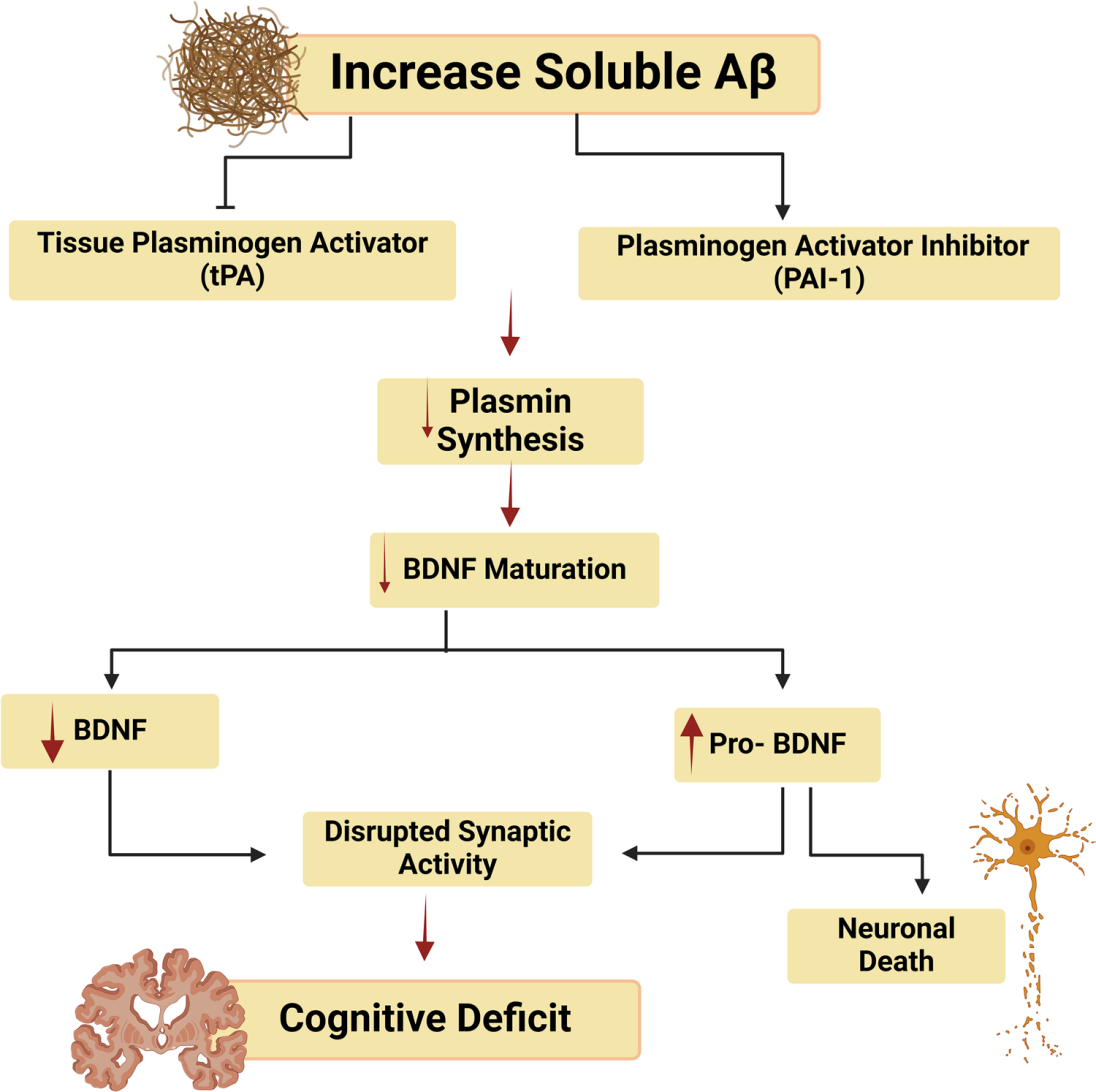
Impairment of BDNF in AD: increase of soluble Aβ inhibits tissue plasminogen activator (tPA) and activates plasminogen activator inhibitor 1 (PAI-1) leading to the reduction of plasmin synthesis and the formation of BDNF. These alterations result in the reduction the formation of BDNF and increasing of proBDNF brain levels with subsequent synaptic dysfunction and the development of cognitive impairment in AD

These findings indicated that BDNF signaling is deregulated in AD, leading to cognitive impairment and memory loss (Fig. 3). Therefore, BDNF signaling stimulation may represent a viable therapeutic approach for AD management.

## BDNF Activators in AD

Importantly, BDNF has a short half-life, cannot cross BBB, and has a potential adverse effect that limits clinical use (Langhnoja, 2018). Because of the BBB, the BDNF must be infused directly, produced by viral constructs, secreted from implanted protein-secreting cells, or transported across the brain. An alternative to this is using a small molecule agonist, a modulator, or an enhancer targeting the associated receptors (Langhnoja, 2018). In Aβ-transgenic mice, *BDNF* gene delivery reverses synapse loss, improves cell signaling, and restores learning and memory. These outcomes occur independently of effects on amyloid plaque load. In aged rats, BDNF infusion reverses cognitive decline, improves age-related perturbations in gene expression, and restores cell signaling. Thus, BDNF exerts substantial protective effects on crucial neuronal circuitry involved in AD, acting through amyloid-independent mechanisms (Nagahara et al., 2009). Unfortunately, most proteins cannot be effectively delivered into the brain from the bloodstream due to the BBB. However, BDNF deliver into the brain of transgenic APP/PS1 mice using ADTC5 as BBB modulator in a mouse model for AD. There were high plaque loads in all groups of mice, suggesting no influence of BDNF on plaque formation. In summary, ADTC5 can deliver BDNF into the brains of APP/PS1 mice, and the activity of BDNF in improving cognitive function was likely due to improvement in synaptic plasticity but not by reducing the plaque load (Kopec et al., 2020). Therefore, direct administration of BDNF in AD patients seems impracticable, and activation of brain BDNF/TrkB signaling could be more compatible with the management of AD.

BDNF/TrkB signaling is highly reduced in AD, resulting in cognitive impairment and synapse failure (Fan et al., 2020). It has been observed that TrkB deletion exacerbates AD neuropathology in mice by lowering the neuroprotective CREB signaling in early AD (Devi & Ohno, 2015). The calpain pathway mediates Aβ-induced TrkB dysfunction (André Jerónimo-Santos et al., 2015a, 2015b). Consequently, some BDNF/TrkB signaling agonists may enhance AD patients’ cognitive performance. As well, using BDNF mimetics may overcome the BDNF pharmacokinetics. It has been revealed that BDNF activators such as TrkB agonists or BDNF mimetic molecules prevent neurodegeneration in animal models (Al-Kuraishy et al., 2024). However, the significant problem linked with the use of BDNF mimetic or small molecules is the widespread effects of these molecules on different receptor subtypes (Al-Kuraishy et al., 2024; Alrouji et al., 2023). BDNF possesses diverse biological functions mediated by the activation of TrkB and the p75^NTR^. The therapeutic potential of BDNF has drawn attention since dysregulation of its signaling cascades has been suggested to underlie the pathogenesis of AD. Multiple strategies targeting BDNF have been tested; most have found obstacles that ultimately hampered its effectiveness (Al-Kuraishy et al., 2024; Alrouji et al., 2023). Extensive research has been directed to assess the therapeutic potential of BDNF in a wide range of brain pathologies, showing auspicious results.

It has been recognized that TrkB agonists such as 7,8-dihydroxyflavone can reverse cognitive dysfunction by inhibiting APP secretase and augmenting endogenous BDNF signaling in transgenic mice (Ghadernezhad et al. 2016). In addition, a synthetic modification of the 7,8-dihydroxyflavone CF3CN compound has a potent neuroprotective effect against the AD mouse model by the exact mechanism (Liu et al., 2010). TrkB receptors are activated when 7,8-dihydroxyflavone is taken orally, according to a pre-clinical investigation, and improves the cognitive functions in rodents by reducing cortical hippocampal Aβ (Liu et al., 2010). A synthetic prodrug of 7,8-dihydroxyflavone known as R13 enhances TrkB signaling and could be effective against AD neuropathology (Chen et al., 2018a). It has been shown that TrkB agonist LMDS-1 protects hippocampal neurons from Aβ in mice by upregulating CREB signaling. In addition, LMDS-1 is more potent than 7,8-dihydroxyflavone in improving BDNF/TrkB signaling (Fan et al., 2020). LM-031, a coumarin derivative, promotes CREB/BDNF signaling and prevents Aβ-neurotoxicity in SH-SY5Y cells via activating the TrkB receptor (Chiu et al., 2022). A full TrkB receptor agonist, ZEB85, improves hippocampal synaptic plasticity and attenuates Aβ-neurotoxicity in primary hippocampal neurons (Tacke et al., 2022). Interestingly, LM22a-4, a small molecule mimicking BDNF, has 85% similarity with BDNF and improves hippocampus neuron survival, while preventing neuronal death in AD models (Massa et al., 2010). Pre-clinical studies highlighted the efficacy of small molecule mimetics of BDNF, such as deoxygedunin and N-acetyl-serotonin, against AD neuropathology (Jang et al., 2010a, 2010b; Jang et al., 2010a, 2010b). Chen et al. (2018b) demonstrated how activation of TrkB signaling in AD-like disease in a rat model by deoxygedunin reduces D-galactose-induced Aβ buildup in the hippocampus and related oxidative stress and cognitive impairment. Similarly, N-acetyl-serotonin activates TrkB/BDNF/CREB signaling in primary neurons to reduce oxidative stress and associated neuronal death (Al-Kuraishy & Al-Gareeb, 2020; Al-Kuraishy et al., 2022a, 2022b, 2022c; Al-Thomali et al., 2022; Mostafa-Hedeab et al., 2022; Sindhu et al., 2021; Yoo et al., 2017). Furthermore, peptide BDNF tetrapeptides have neuroprotective properties in primary neurons by stimulating TrkB signaling. Of interest is that the B5 peptide triggers the expression of TrkB receptors; however, B3 increases the action of BDNF on TrkB receptors, which enhances the survival of hippocampus nerves. BDNF tetrapeptides are the partial agonists of TrkB receptors that can reverse neurodegeneration in hippocampal neurons in animal models (Cardenas-Aguayo et al., 2013). Therefore, BDNF tetrapeptides have a specific neuroprotective effect when BDNF is reduced, as in AD. In addition, peptide 021 (P021) ameliorates the cognitive abilities in transgenic mice by enhancing the hippocampus neurogenesis and synaptic plasticity by increasing the expression of BDNF (Li et al., 2010). However, P021 was not evaluated by pre-clinical studies.

Moreover, C31, a small non-peptide mimetic molecule that activates p75NTR, has a neuroprotective effect in early AD. C31 attenuates Aβ-induced neurotoxicity by activating the neuroprotective signaling. C31 prevents AD-like pathology in transgenic mice and significantly protects against AD in Phase II clinical trials (Longo & Massa, 2013). These findings indicated that TrkB agonists and BDNF mimetic peptides could be effective against AD neuropathology by increasing brain BDNF signaling. However, these agents were tested in pre-clinical studies, and most failed to be completed in the clinical trials (Longo & Massa, 2013).

In this manner, searching for approved drugs that enhance brain BDNF might be helpful in AD management.

By blocking 3-hydroxy-3-methyl glutaryl coenzyme A reductase (HMG-CoA), a rate-limiting enzyme in producing hepatic cholesterol, statins are medications that reduce cholesterol. Statins are used to manage dyslipidemia and hypercholesterolemia and to avoid the secondary consequences of cardiovascular diseases (Al-Kuraishy et al., 2021, 2020a, 2020b, 2020c). Statins are also effective in the prevention of neurodegenerative diseases (Al‐kuraishy et al., 2023). Studies showed that statins efficiently manage AD (Alsubaie et al., 2022; Wełniak et al., 2020). There is a contentious issue regarding the impact of statins on cognitive function in AD (Alsubaie et al., 2022). A meta-analysis and comprehensive review found no evidence linking statins to cognitive impairment, as well, statins have favorable effects against AD neuropathology (Olmastroni et al., 2022). Statins have been shown in numerous studies to enhance brain BDNF by different mechanisms, including phosphorylation of Akt, glycogen synthase kinase-3β (GSK-3β), and cAMP-response element-binding proteins (CREB) (Tsai, 2007; Zhang et al., 2017). A case–control investigation including 78 ischemic stroke patients showed that lipid-soluble atorvastatin improves the clinical outcomes of stroke patients by increasing plasma BDNF levels compared to the placebo (Zhang et al., 2017). According to Tsai’s research (Tsai, 2007), statins facilitate the proteolytic degradation of proBDNF by blocking plasminogen activator inhibitor 1, a significant plasmin inhibitor that enables the conversion of proBDNF to BDNF. Besides, pitavastatin improves BDNF/TrkB signaling in the primary cerebral neurons (Cui et al., 2018). Simvastatin enhances recovery of function after experimental spinal cord damage by upregulating BDNF expression, as demonstrated by Han et al. (Han et al., 2011). These decisions suggested that statins can improve AD neuropathology by upregulating BDNF/TrkB signaling and its expression.

Metformin is an insulin-sensitizing medication used to treat type 2 diabetes. Metformin has pleiotropic properties, including antioxidant and anti-inflammatory properties (H. Al-Kuraishy et al., 2019, 2023; Al‐kuraishy et al., 2022). Furthermore, by mitigating brain signaling and reducing brain insulin resistance, metformin can reduce the risk of different neurodegenerative diseases (Al-Kuraishy et al., 2023d; Alnaaim et al., 2023; Alrouji et al., 2024). A pilot study found that metformin improves cognitive function in AD patients (Gupta et al., 2011). Pre-clinical studies highlighted that metformin prevents the production of Aβ and reduces the hyperphosphorylated tau protein level in neuroblastoma cell lines and primary neurons, respectively (Kickstein et al., 2010).

In the same way, metformin decreases tau protein and APP in the AD model to enhance cognitive performance (Farr et al., 2019). Moreover, many studies showed that metformin improves the expression of BDNF by increasing the histone acetylation along with the BDNF promoter, which was attributed to the activation of AMPK and CREB (Ameen et al., 2022; Fang et al., 2020; Ghadernezhad et al., 2016). It has been illustrated that metformin recovers cognitive function by increasing BDNF expression in the forebrain in ischemic rats (Fang et al., 2020). As well, metformin alleviates stress-induced depression in mice by enhancing BDNF signaling (Fang et al., 2020). Furthermore, metformin improves the neurocognitive function in aged rats by enhancing the signaling pathway of BDNF/PI3K (Ameen et al., 2022). Thus, metformin seems to possess a neuroprotective impact against the neuropathology of AD by BDNF signaling activation.

## Limitations and Challenges of BDNF Activators in AD

Nevertheless, many limitations were recognized, especially regarding its delivery and central availability. However, novel developments have been made to tackle this issue through various approaches, such as gene therapy and carrier-free stabilizing nano-encapsulation, which allows intranasal administration. Other techniques include using TrkB receptor ligands such as BDNF mimetics and agonistic antibodies and compounds boosting BDNF synthesis, transmission, and signaling, including natural products. However, these direct and indirect approaches, referred to as BDNF-based therapies, still face many other challenges. Moreover, further research is still required to elucidate the network-specific functionality of BDNF, decipher how this modulates cognition and behavior, and uncover BDNF transmission disruptions in a disease-specific manner. Other considerations include systemic side effects, interactions with other disorders in which elevated BDNF levels can have harmful long-term consequences, and the potential impact of different polymorphisms on the efficacy of BDNF-based therapies (Miranda-Lourenço et al., 2020). BDNF has been proven to improve functional recovery in pre-clinical and, to a lesser extent, clinical studies. Direct and indirect methods to increase levels of neurotrophic factors in animal models have successfully improved post-injury outcome measures. However, the translation of these studies into clinical trials has been limited. Pre-clinical experiments have largely failed to impact clinical research [94] significantly. Due to its critical and very pleiotropic activity, reduction of BDNF levels and alterations in the BDNF/TrkB signaling are connected with a broad spectrum of neurological diseases. Nonetheless, because of its poor bioavailability and pharmacological properties, BDNF itself has a very low therapeutic value (Bazzari & Bazzari, 2022). To overcome this obstacle, different approaches were suggested to enhance the delivery of the BDNF, such as intranasal route and nanotechnology nano-carrier across the BBB (Padmakumar et al., 2021; Pilakka-Kanthikeel et al., 2013).

Moreover, the concomitant binding of exogenous BDNF to the p75^NTR^ can elicit several unwanted and deleterious side effects (Azman & Zakaria, 2022a, 2022b). Furthermore, TrkB antibodies have been shown to bind the TrkB, not p75NTR specifically. Thus, while, based on the current knowledge, the TrkB agonists do not seem to have the potential to reverse the disease pathology per se, promoting BDNF/TrkB signaling still has a very high therapeutic relevance (Azman & Zakaria, 2022a, 2022b; Bazzari & Bazzari, 2022). Several attempts have been made to evade the limitations to the therapeutic use of recombinant BDNF and to develop drugs with a higher specificity for the BDNF/TrkB signaling. These include the development of a series of low molecular weight drugs with more favorable pharmacokinetic properties and a high degree of specificity for the TrkB receptor and TrkB agonist antibodies. Therefore, the results obtained with several non-peptide small molecule TrkB agonists should be deduced with caution, keeping in mind that some of the beneficial effects observed could be due to TrkB-independent neuroprotective and effects as shown for 7,8-DHF due to its antioxidant activity (Liu et al., 2010). Therefore, developing specific TrkB agonists with robust activity in both in vitro and in vivo systems remains an important goal.

Moreover, the poor pharmacokinetic properties and lack of observed activation of TrkB-dependent signaling in the brain confirmed that 7,8-DHF is not a relevant tool for studying TrkB activation in vivo*,* suggesting a distinct functional profile independent of interaction with TrkB (Pankiewicz et al., 2021). The activity of 7,8-DHF in vivo is not mediated through direct TrkB activation. Still, it can be attributed to other molecular targets that the molecule or its derivatives may activate. In addition, allosteric interaction with TrkB offers new opportunities, and a shift toward the crossed transmembrane domain of the TrkB receptor as a target for novel agonist pharmacophores should be considered in future development engaging the BDNF/TrkB signaling pathway (Pankiewicz et al., 2021). Therefore, 7,8-DHF should be carefully considered as a reference compound for a TrkB receptor agonist and BDNF-mimicking agent in further analysis. While the beneficial effects of treatment with TrkB agonist drugs are apparent for very diverse neurological diseases, whether these drugs have the potential to become a disease-modifying treatment is still open. On the other hand, whether the alterations in BDNF/TrkB signaling described are the cause of the disease or rather a correlation or even a secondary consequence of the cell loss is still unclear.

Additionally, BDNF/TrkB signaling activators such as TrkB receptor agonists and BDNF mimetics are associated with different adverse effects. Mounting evidence suggests a bi-directional connection between BDNF expression and regulation of inflammation. Increased production of BDNF from immune cells, including infiltrating T-cells and macrophages, contributes to the development of inflammation during allergic asthma, promoting neuronal changes leading to airway smooth muscle contraction and mucus hypersecretion (Braun et al., 2004). Moreover, the pathology of all neurodegenerative diseases, as well as other neurological conditions, is characterized by chronic neuroinflammation (Lima Giacobbo et al., 2019). Indeed, microglia, monocytes, and astrocytes secrete BDNF in response to TNF-α and IL-6, and in turn, BDNF levels modulate microglial proliferation and activation. Meanwhile, increased BDNF levels promote microglial proliferation and activation during immune challenges (Lima Giacobbo et al., 2019). In addition, overexpression of brain BDNF is linked with the development of epilepsy by inducing neuronal hyper-excitability; BDNF serum level is correlated with the severity of epileptic seizures (McGonigal et al., 2023). However, TrkB receptor agonists attenuate the development and progression of epilepsy through a BDNF-independent pathway (Kipnis et al., 2020). These possible adverse effects may limit the therapeutic potential of BDNF/TrkB signaling activators in AD.

**Fig. 4:**
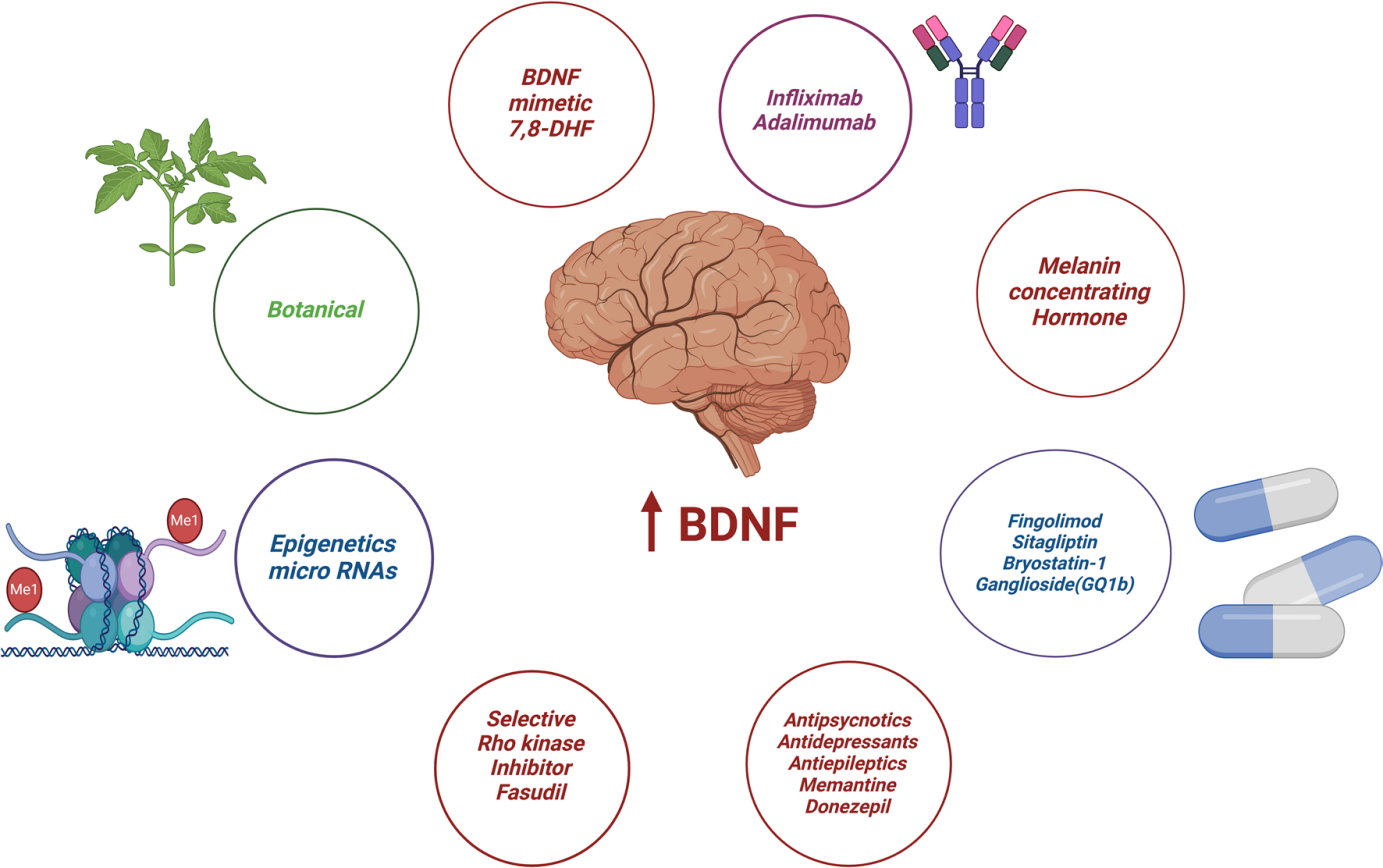
Agents that improve BDNF expression: synthetic drugs and botanical agents as well as epigenetic factors enhance the expression of central BDNF

Taken together, activators of BDNF signaling have crucial roles in mitigating memory and cognitive impairment in AD. Therefore, repurposing commonly approved drugs that can improve brain BDNF signaling might be a useful therapeutic approach for AD management (Fig. 4).

## Conclusions

AD is an aging-related chronic illness that causes memory loss and cognitive impairment due to progressive degeneration of brain neurons, mainly in the cerebral cortex and hippocampal regions. A particular protein called BDNF is essential to several CNS processes, including neurogenesis and plasticity of synapses. BDNF exerts its biological function via activation of TrkB. Dysregulation of BDNF signaling is implicated in the pathogenesis of AD. BDNF/TrkB signaling is highly reduced in AD, leading to synaptic failure and cognitive impairment. The level of serum BDNF is thought to be a possible biomarker of neuropathology of AD. BDNF signaling is deregulated in AD, resulting in cognitive impairment and loss of memory. Consequently, BDNF signaling activation may be a potent therapeutic approach for AD management. Prominently, BDNF has a short half-life, cannot cross BBB, and has a potential adverse effect that limits its use clinically. These findings indicated that TrkB agonists and BDNF mimetic peptides could be efficient against the neuropathology of AD by increasing brain BDNF signaling. However, these agents were tested in pre-clinical studies, and most failed to be completed in the clinical trials. In this way, searching for approved drugs that enhance brain BDNF could effectively manage AD. Both statins and metformin have a neuroprotective effect against AD neuropathology through activation of BDNF signaling. Collectively, activating BDNF signaling has potential roles in alleviating AD’s impairment of memory and cognition. Consequently, repurposing commonly approved drugs that can improve brain BDNF signaling could be an effective therapeutic strategy in the management of AD.

## Data Availability

No datasets were generated or analyzed during the current study.
